# Infertility and Auto-Antibodies: A Review

**DOI:** 10.3390/antib14030076

**Published:** 2025-09-05

**Authors:** Brigita Šemeklienė, Brigita Gradauskienė

**Affiliations:** Department of Immunology and Allergology, Medical Academy, Lithuanian University of Health Sciences, 44037 Kaunas, Lithuania

**Keywords:** infertility, antibodies, immune cells, autoimmunity

## Abstract

Infertility is a multifactorial condition with a wide range of potential causes, including anatomical, hormonal, genetic, and lifestyle-related factors. Among these, immunological mechanisms have increasingly been recognized as important contributors. The immune system plays a critical role in maintaining reproductive health, and its dysregulation can impair fertility in both men and women. Recent scientific studies suggest that altered immune responses, particularly those involving autoimmune reactions, may negatively affect fertility by disrupting the complex immunological balance required for successful conception and pregnancy maintenance. This review focuses on the most common autoantibodies, such as antinuclear, antisperm, antiendometrial, antiovarian, antiphospholipid, and antithyroid antibodies. Treatment options, including immunomodulatory therapy, hormone replacement therapy, and lifestyle interventions, are also reviewed.

## 1. Introduction

Infertility, defined as the inability to achieve pregnancy after 12 months of regular unprotected intercourse (or after 6 months in women over 35), affects an estimated 10–15% of couples worldwide [1]. As a complex and multifactorial condition, infertility encompasses anatomical, endocrine, genetic, environmental, and, increasingly recognized, immunological causes. While traditional diagnostics often prioritize tubal, ovulatory, or male-factor abnormalities, growing evidence underscores the substantial role of autoimmune processes in reproductive dysfunction [2].

Autoimmune infertility refers to a state where immune dysregulation leads to the formation of autoantibodies that interfere with the reproductive system. These antibodies may target various structures and molecules, including the endometrium, ovaries, spermatozoa, and phospholipids. Among these, the presence of antinuclear antibodies (ANA), antiovarian antibodies (AOA), antisperm antibodies (ASA), antiphospholipid antibodies (APL), antithyroid antibodies, and antiendometrial antibodies (AEA) has been associated with impaired fertility outcomes in both natural and assisted conception cycles [3,4].

One of the best-established links between autoimmunity and reproductive failure is seen in antiphospholipid syndrome (APS). Women with APS are prone to recurrent pregnancy loss, preeclampsia, placental insufficiency, and implantation failure, due to the thrombogenic and inflammatory environment induced by APL [5]. Likewise, thyroid autoimmunity—even in euthyroid women—has been correlated with reduced conception rates and increased miscarriage risk, likely due to immune-mediated disruption of the ovaries and endometrium [6,7].

In men, ASA have been shown to impair sperm motility and fertilizing capacity, contributing to unexplained male infertility. Although some studies dispute the clinical relevance of ASA in assisted reproductive technologies (ART) settings, others support their detrimental impact, especially in natural conception or intrauterine insemination attempts [8,9].

The pathophysiological mechanisms linking autoantibodies to infertility remain diverse. Autoantibodies can initiate complement activation, cytokine release, and local inflammation, ultimately damaging reproductive tissues or impairing implantation [10,11]. Furthermore, immune-mediated hormonal dysregulation may alter ovarian function, folliculogenesis, or luteal support.

Despite these associations, autoimmune infertility remains underdiagnosed, partly due to limited awareness and the absence of standardized screening protocols. However, the diagnostic value of antibody testing—especially ANA, AOA, and APL—has been increasingly supported in the work-up of unexplained infertility and recurrent implantation failure [12,13].

Given the rising prevalence of autoimmune diseases in reproductive-age individuals and the impact of delayed childbearing, understanding the immunological dimensions of infertility is becoming ever more pertinent. This review aims to synthesize current evidence regarding key autoantibodies implicated in infertility, elucidate potential mechanisms, and evaluate available diagnostic and therapeutic strategies.

## 2. Methods

This review was conducted with the aim of synthesizing current evidence on the relationship between autoantibodies and infertility. A comprehensive search of the biomedical literature was carried out using three major electronic databases: PubMed, ScienceDirect, and Google Scholar. The search strategy included a combination of keywords and medical subject terms such as “infertility,” “autoantibodies,” “autoimmunity,” “antinuclear antibodies,” “antisperm antibodies,” “antiendometrial antibodies,” “antiovarian antibodies,” “antiphospholipid antibodies,” and “thyroid autoantibodies.” Boolean operators were employed to refine and expand search results where appropriate. The search was limited to articles published in English between January 2010 and April 2025.

The review focused on selecting peer-reviewed original research articles, clinical studies, systematic reviews, and meta-analyses that investigated the role of autoantibodies in either male or female infertility. Studies were included if they addressed immunological mechanisms, diagnostic applications, or therapeutic implications of autoantibodies in the context of infertility. Non-English publications, animal studies not directly translatable to human reproduction, editorials, and conference abstracts lacking primary data were excluded from the analysis.

**Figure 1:**
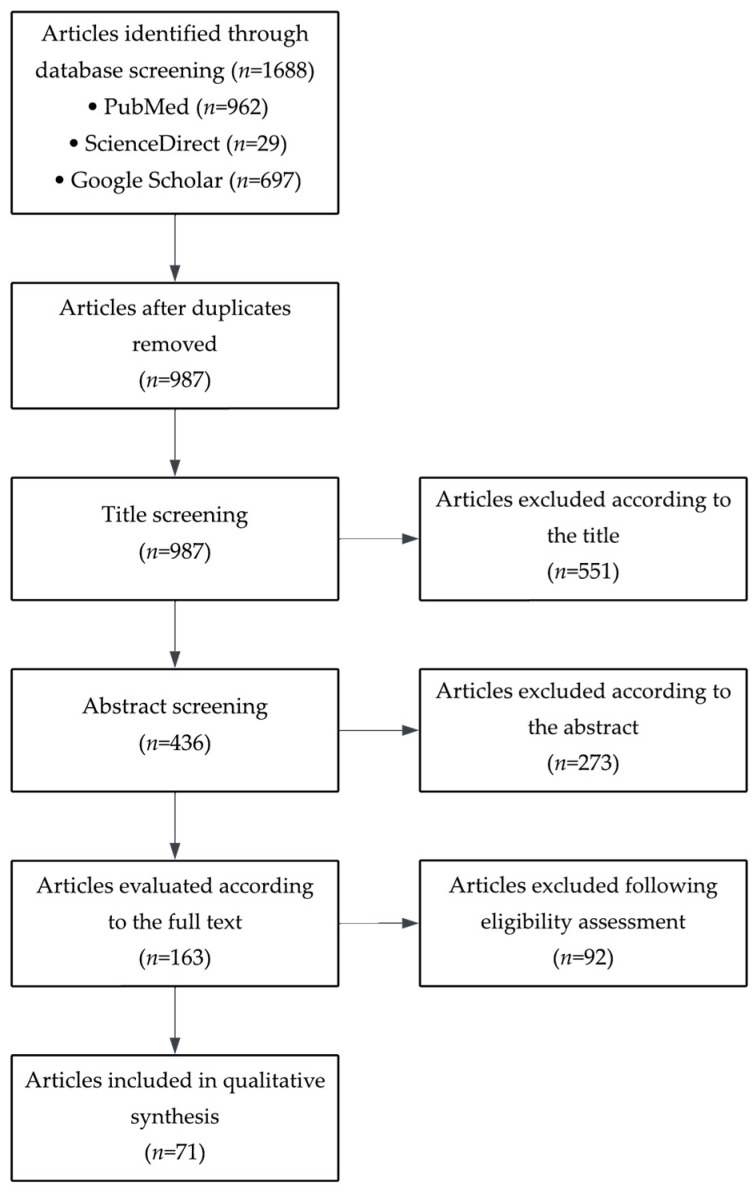
Flowchart of the selection of the different studies used in this article.

Initial screening of titles and abstracts was performed to identify potentially relevant articles. Full texts of eligible studies were subsequently retrieved and reviewed in detail. The selection process involved two independent reviewers who assessed the methodological relevance and scientific quality of each article. In cases of disagreement, consensus was reached through discussion. From the selected studies, key information was extracted, including study design, participant characteristics, types of autoantibodies investigated, diagnostic techniques used, and reproductive outcomes assessed. Particular attention was given to outcomes such as conception rates, success of assisted reproductive technologies, oocyte and sperm quality, miscarriage rates, and the presence of immunological or hormonal disturbances. Detailed selection of different references used in this article is shown in a flowchart (Figure 1).

The extracted data were analyzed using thematic synthesis, with studies grouped according to the type of autoantibody examined. Recurring patterns and associations were identified across different categories, such as antinuclear, antisperm, antiendometrial, antiovarian, antiphospholipid, and antithyroid antibodies. The review also considered studies that investigated the effectiveness of immunomodulatory or hormone-based treatments in patients with identified autoantibodies.

To ensure a high level of reliability in the evidence presented, preference was given to studies with clearly described methodologies, appropriate control groups, and adequate sample sizes. Particular emphasis was placed on research that demonstrated direct clinical relevance to the diagnosis or treatment of autoimmune-related infertility. The final synthesis aimed to provide a structured overview of the immunological factors contributing to infertility and to highlight potential diagnostic and therapeutic strategies based on current scientific knowledge. A shortened version of found results is shown in Table 1.

## 3. Antinuclear Antibodies

The relationship between antinuclear antibodies (ANA) and infertility is a subject of significant interest in reproductive immunology. ANA are autoantibodies that target the nucleus of cells and are often associated with various autoimmune diseases. Their presence has been linked to several reproductive challenges, including infertility and adverse outcomes in assisted reproductive technologies such as in vitro fertilization (IVF).

Studies indicate that the presence of ANA can adversely affect reproductive outcomes. Wu et al. reported that ANA in follicular fluid might be a risk factor for IVF and embryo transfer [14]. Additionally, Mitić et al. found that treatment with dexamethasone and aspirin improved IVF outcomes in women with positive autoantibody tests, emphasizing that autoimmune dysregulation could directly impact fertility [15]. Similarly, Guo et al. demonstrated that hydroxychloroquine could enhance pregnancy outcomes in patients undergoing frozen embryo transfers with positive autoantibody tests, suggesting a significant connection between autoimmunity and reproductive success [4]. However, it should be noted that some studies have reported conflicting results regarding the association between ANA and adverse reproductive outcomes, indicating that more research is needed to clarify these relationships.

A meta-analysis conducted by Chen et al. corroborated these findings by examining the association between ANA and infertility, indicating a link between elevated ANA levels and an increased prevalence of infertility, with specific mentions of diminished oocyte quality and higher rates of miscarriage and IVF failure [3,16].

The clinical manifestation of infertility in individuals with elevated ANA levels can also be attributed to immunological alterations affecting implantation and embryo development. Findings from Li et al. suggested that the presence of ANA in follicular fluid might impede the efficacy of IVF by affecting the endometrium and granulosa cell function [10]. Such interference may lead to reduced implantation rates and consequently lower pregnancy rates in individuals undergoing assisted reproductive techniques.

Autoimmune diseases, including systemic lupus erythematosus, have been documented to negatively influence fertility outcomes. Research indicates that women diagnosed with systemic lupus erythematosus often present with elevated levels of various autoantibodies, including ANA, which can complicate their reproductive health, correlating with difficulties in achieving conception [17]. This is compounded by findings that some autoimmune conditions might contribute indirectly to ovarian reserve depletion [42].

Additionally, the literature points to a significant prevalence of autoantibodies among women with unexplained infertility. A review by Koshak et al. indicated that patients with unexplained infertility often display higher levels of ANA, suggesting that a subset of these women may be experiencing immunological barriers to conception due to underlying autoimmune issues [2]. This aligns with studies that report autoimmune infertility manifesting as recurrent implantation failure or unexplained infertility [12].

Moreover, the interplay between ANA and other autoantibodies cannot be overlooked. A comprehensive review by Cavalcante et al. noted that other autoantibodies, such as APL, can exacerbate infertility issues through their impact on reproductive functioning [13]. The relationship between these autoantibodies and reproductive health underscores the importance of thorough immunological evaluations in women facing infertility.

The mechanism by which ANA may contribute to infertility typically involves the disruption of trophoblast cell function and the necessary endocrinological adaptations needed during pregnancy. Some studies have shown that ANA can interfere with trophoblast proliferation and function, which are essential for successful embryo implantation [43]. Furthermore, autoimmunity may influence inflammatory cytokine environments, creating conditions unfriendly to embryo implantation [44].

Research into treatment strategies for women with ANA reveals a growing interest in immunomodulatory therapies aimed at improving reproductive outcomes. Treatments such as prednisolone and aspirin have reportedly improved IVF outcomes in women with autoimmune conditions, suggesting potential therapeutic avenues for this patient group [45,46]. Furthermore, integrating hydroxychloroquine into treatment regimens has emerged as a promising option, as its immunomodulatory properties have been associated with enhanced pregnancy rates in women with positive autoantibody tests [4].

Evidence underscores how the presence of ANA correlates with infertility, potentially through mechanisms of impaired oocyte quality, embryo development, and inflammatory responses that hinder implantation. Ongoing research aimed at elucidating these relationships continues to reveal the complex interplay between autoimmunity and reproductive health, warranting heightened attention to the immunologic status of women undergoing fertility treatments.

## 4. Antisperm Antibodies

Antisperm antibodies (ASA) are a group of immunoglobulins produced by the immune system that target sperm antigens. The development of these antibodies is often linked to various pathological conditions affecting male fertility, leading to a form of infertility known as immune infertility. Antisperm antibodies can adversely influence numerous sperm functions, thereby hindering male reproductive potential. As such, the relationship between ASA and infertility is a critical area of research in reproductive healthcare.

Antisperm antibodies are known to perform several detrimental functions, including immobilizing sperm, agglutinating them, and interfering with fertilization processes. Studies indicate that the presence of ASA is associated with a decrease in fertility rates among men. For instance, Sciorio et al. elucidated how inflammation and autoimmunity could lead to the production of ASA, recognizing sperm as foreign entities and activating an immune response, contributing to infertility [18]. Furthermore, a systematic review by Silva et al. highlighted that ASA are present in 5–15% of men exhibiting fertility issues, thereby underscoring their significance in reproductive dysfunction [8].

The prevalence of ASA is notably higher in specific populations, such as men with varicocele, a condition associated with impaired venous drainage from the testis. Research by Bozhedomov et al. established a strong correlation between the presence of ASA and transplantation success rates post-microsurgical varicocelectomy [19]. This suggests that the potential for antisperm immunity increases in cases where the blood-testis barrier is compromised, resulting in the exposure of sperm antigens to the immune system and subsequent antibody production [8].

Moreover, patients who have undergone surgical procedures such as vasectomy are also at risk for the development of ASA post-reversal procedures. For instance, Wang et al. found that the presence of ASA can persist long after surgical interventions like vasectomy and may lead to autoimmune infertility [20]. Such findings emphasize the complex interplay between surgical history and autoimmune fertility challenges.

Immunological infertility due to ASA can take various forms. They can impede natural fertilization by obstructing sperm motility, preventing sperm from successfully navigating through the female reproductive tract. Gizzo et al. indicated that the presence of human papillomaviruses deoxyribonucleic acid (HPV-DNA) in sperm could co-occur with ASA, leading to further complications in male fertility [47]. Furthermore, these autoantibodies can also hinder sperm capacitation and the acrosome reaction, which are crucial processes for successful fertilization [48].

In terms of diagnostic measures, testing for ASA is essential in evaluating male infertility. Utilizing methods such as the mixed antiglobulin reaction test enables clinicians to identify the presence and levels of ASA in seminal fluid, guiding treatment decisions [49]. Moreover, research shows that men with detectable ASA may benefit from utilizing assisted reproductive technologies like intracytoplasmic sperm injection, which can bypass some barriers that ASA create in natural fertilization processes [9].

While many studies suggest a negative impact of ASA on fertility outcomes, the evidence is not universally conclusive. Some researchers argue that the presence of ASA does not always correlate with significant declines in reproductive success, indicating other underlying factors may contribute to infertility cases [8]. Moreover, a systematic review highlighted that the relationship between ASA and infertility varies significantly, suggesting the need for further research to elucidate this complex interplay [22].

Emphasizing the importance of lifestyle factors, Kamphorst et al. mentioned the influence of environmental exposures on the prevalence of ASA, with factors such as infection and inflammation contributing to increased antibody production [21]. Therefore, improving general health and reducing exposure to environmental toxins may also serve as potential strategies in managing ASA-related infertility.

Antisperm antibodies represent a significant immunological factor that can contribute to infertility in males. ASA can adversely affect various sperm functions necessary for successful fertilization and can emerge from numerous underlying medical and lifestyle factors. Understanding the mechanisms through which these antibodies operate allows researchers and clinicians to enhance diagnostic and treatment protocols for male infertility, employing strategies like intracytoplasmic sperm injection where applicable.

## 5. Antiendometrial Antibodies

The role of antiendometrial antibodies (AEA) in infertility is a subject of growing interest within reproductive immunology. These antibodies are developed against endometrial tissue, which plays a critical role in implantation and the maintenance of early pregnancy. The presence of AEA has been linked to various reproductive challenges, such as implantation failure and recurrent pregnancy loss. This overview highlights the mechanisms through which AEA may impact fertility and the implications for identifying and managing reproductive issues in affected individuals.

Bobak et al. indicated that antibodies against the fertilization antigen FA-1 can block sperm binding to the oocyte, which may represent a mechanism of failure in both natural fertilization and intrauterine insemination [25]. Here, it is evident that the immune response evoked by AEA might extend beyond just the endometrial environment to include a functional impairment in gamete interaction, ultimately affecting fertility outcomes.

The presence of AEA can further exacerbate conditions such as endometriosis, where the immune system may view endometrial-like tissue located outside the uterus as foreign, leading to an autoimmune response. The complex interplay between autoimmunity and fertility has been explored by various researchers. It is noted that infections, such as Chlamydia trachomatis, may elicit immunological responses leading to infertility by activating autoantibodies, including AEA [26]. Such interactions complicate the clinical picture, indicating that checking for AEA may be particularly essential in women with a history of endometriosis or chlamydia infections.

In considering the pathophysiological implications of AEA, studies have shown that such antibodies can disrupt the normal microenvironment of the endometrium. Specifically, anti-laminin-1 antibodies have been associated with altered endometrial receptivity, highlighted in work by Caccavo et al. [27]. These alterations not only cause premature ovarian insufficiency and affect the implantation process but may also contribute to an inappropriate immune profile during the crucial implantation window.

The detection of these antibodies remains a challenge; however, advancements have been made in establishing protocols for their diagnosis. For instance, while specific tests for AEA have been suggested, including enzyme-linked immunosorbent assays (ELISA), literature on their reliability reveals varying degrees of sensitivity and specificity [50]. The ability to track these antibodies clinically could facilitate tailored interventions for women suffering from infertility attributable to autoimmune mechanisms.

Therapeutic approaches for managing infertility associated with AEA are still evolving. Existing literature indicates that treatments often focus on modulating the immune response, such as utilizing corticosteroids or other immunosuppressive agents to reduce the effects of these antibodies on reproductive functions [51]. However, the effectiveness of such interventions remains inconsistent, prompting the need for further research to validate their efficacy and establish standardized treatment protocols.

Additionally, multidisciplinary approaches involving both reproductive endocrinologists and immunologists could optimize management strategies for women with elevated AEA, refining diagnostic criteria and therapeutic options alike. Integrating these perspectives may also facilitate advancements in ART protocols, benefiting those facing infertility due to antiendometrial immunity.

Antiendometrial antibodies represent a critical area of inquiry within reproductive immunology, with their presence posing substantial challenges to fertility, particularly in women with conditions like endometriosis. Understanding the mechanisms through which AEA operate, alongside improved diagnostic and therapeutic strategies, can enhance the care provided to individuals suffering from immunologically mediated infertility.

## 6. Antiovarian Antibodies

Antiovarian antibodies (AOA) are autoantibodies that target ovarian tissues and antigens, potentially disrupting normal ovarian function and fertility. The presence of these antibodies has been increasingly recognized as a factor contributing to female infertility, particularly in the context of autoimmune disorders. Understanding the mechanisms through which AOA operate and their implications for fertility is critical for effective diagnosis and treatment in affected individuals.

Several research studies have identified a correlation between the presence of AOA and conditions such as premature ovarian insufficiency and unexplained infertility. Luborsky et al. identified anti-ovarian antibodies in women with premature ovarian insufficiency, suggesting a potential role in ovarian dysfunction [28]. These autoantibodies can interfere with hormonal signaling and disrupt normal ovarian function, leading to inadequate follicular development and ovulation, a scenario that plays a crucial role in achieving pregnancy.

Moreover, the dysregulation of immune responses, often associated with the production of AOA, has been described as contributing to fertility challenges in women. Müller et al. suggested that reduced fecundity might be associated with the dysregulation of immune system reactions resulting in enhanced autoantibody production, including AOA [29]. This suggests that women with a history of autoimmunity may be predisposed to develop these antibodies, subsequently affecting their reproductive health.

The role of AOA can also be associated within the larger context of immunological infertility. Research has indicated that these antibodies may play a role in blocking the interaction between oocytes and sperm, thereby hindering fertilization. Abdullah reported that autoimmune factors, including AOA, could account for a significant proportion of infertility cases, as they may disrupt critical reproductive processes [30].

A noteworthy interaction exists between AOA and hormonal regulation as well. Findings from Khadhim et al. indicated that infertile women with AOA may also display antibodies against follicle-stimulating hormone (FSH), highlighting how the immune response targeting ovarian antigens can lead to various reproductive dysfunctions associated with irregular hormone production [31].

The detection and diagnosis of AOA involve serological testing, which can be challenging due to varying techniques and limited standardization. According to Chen, identifying specific autoantibody profiles can assist in diagnosing autoimmune infertility, as elevated levels of both antisperm and AOA have been linked to poorer reproductive outcomes [32]. This dual approach to assessing antibodies could enhance diagnostic accuracy and lead to tailored interventions for improving fertility outcomes in women.

Several therapeutic strategies are currently being explored to address infertility associated with AOA. One approach involves the use of immunomodulatory treatments aimed at reducing the autoimmune response. The use of corticosteroids has been proposed as a method to alter the immune response in women with autoimmune conditions, potentially improving fertility by mitigating the effects of AOA. However, further research is essential to confirm the efficacy of such treatments and to establish guidelines for clinical practice.

The connection between AOA and polycystic ovarian syndrome (PCOS) has also garnered attention in the literature. Abood and Hathal discussed correlations between the presence of AOA and the clinical manifestations of PCOS, suggesting that autoimmune mechanisms may play a role in the pathology of the syndrome [33]. This intersection of autoimmune activity and hormonal irregularities in PCOS patients raises questions about how to best evaluate and manage fertility in these populations.

Behavioral and lifestyle factors may also influence the expression and effects of AOA. Chronic inflammatory conditions, such as those associated with obesity, have been found to increase the likelihood of developing autoantibodies in several studies. This highlights the need for comprehensive assessments that take into consideration metabolic health alongside immunological evaluations to better identify treatment options for infertility.

Antiovarian antibodies play a significant role in female infertility, with their presence directly correlating with various reproductive dysfunctions, including issues with ovulation and fertilization. Increasing awareness around the influence of these antibodies on reproductive health can be pivotal for clinicians seeking to support women facing infertility. Moving forward, standardized diagnostic tests, improved therapeutic options, and further research into the mechanisms behind autoimmunity in reproductive health will be essential for effectively managing infertility associated with AOA.

## 7. Antiphospholipid Antibodies

Antiphospholipid antibodies are a heterogeneous group of autoantibodies that target phospholipid-binding proteins, and they are classically associated with an autoimmune condition known as antiphospholipid syndrome (APS). The significance of these antibodies extends beyond their association with thrombotic events; they are implicated in a range of pregnancy complications, including recurrent pregnancy loss, placental insufficiency, and preeclampsia, along with a myriad of adverse obstetric outcomes. This overview elucidates the complex relationship between APL and infertility, highlighting underlying mechanisms, clinical implications, and potential therapeutic strategies.

Firstly, the correlation between APL and infertility primarily manifests through their association with recurrent pregnancy loss. Research has shown that a significant proportion of women with unexplained recurrent pregnancy loss had positive test for APL. According to a study by Kirovakov et al., women with hereditary thrombophilia demonstrated significantly higher levels of APL, correlating with increased incidence of pregnancy loss [34]. This suggests that APL can precipitate early pregnancy complications by promoting thrombosis within the placental vasculature, thus impairing fetal development.

Furthermore, the presence of APL can lead to various obstetric complications by promoting a pro-inflammatory environment within the uterus. The antibodies may activate complement pathways, stimulating local inflammation and subsequently disrupting the delicate processes of implantation and placentation. Research conducted by Poindron et al. emphasizes that the immune response triggered by APL can contribute to both embryonic and placental dysfunction, thus exacerbating risks of miscarriage and fetal demise as well as potential maternal complications during pregnancy [11].

In addition to direct fetal effects, APL can also impact ovarian function. The immunological effects of APL may interfere with folliculogenesis and ovulation, potentially leading to anovulation or suboptimal ovarian response in women attempting conception. This disruption could hinder natural conception or compromise the success of assisted reproductive technologies. For instance, women with APS seeking IVF may be at higher risk for unsuccessful outcomes due to not only the embryonic loss but also potential poor ovarian response attributed to the immune mechanisms associated with APL.

Diagnostics play a critical role in managing infertility related to APL. Testing for these antibodies typically involves assessing levels of anticardiolipin antibodies, lupus anticoagulant, and anti-β2 glycoprotein I antibodies. The diagnosis of APS can be made when at least one of these antibodies is present, alongside clinical or historical evidence of thrombosis or pregnancy morbidity [52]. According to Dayco et al., identifying and managing APS effectively can improve reproductive outcomes by addressing underlying coagulopathies and reducing the risk of miscarriage through appropriate medical interventions [53].

In this context, the recently introduced 2023 ACR/EULAR APS classification criteria provide an updated, weighted scoring system that increases diagnostic specificity to 99% while maintaining good sensitivity. These criteria require at least one positive aPL test within three years of an aPL-associated clinical manifestation, with additive points assigned across six clinical domains (macrovascular venous, macrovascular arterial, microvascular, obstetric, cardiac valve, and hematologic) and two laboratory domains (lupus anticoagulant functional assays and solid-phase IgG/IgM anticardiolipin or anti–β2-glycoprotein I antibodies) [54]. Recognition is also growing for seronegative APS, in which patients meet clinical criteria without conventional laboratory markers, highlighting the need for careful clinical assessment.

Thromboprophylaxis is an essential component of the management strategy for women with APS who are experiencing infertility. Studies have demonstrated that low-dose aspirin and anticoagulant therapy with heparin can significantly reduce the risk of pregnancy complications in these women. A systematic review by Schreiber et al. suggested that these interventions could lead to improved pregnancy outcomes in women with APL who underwent assisted reproductive technology, highlighting the importance of early diagnosis and proactive management [5].

Moreover, adjunct therapies using hydroxychloroquine have also emerged as promising treatment strategies. Hydroxychloroquine may help regulate immune dysregulation associated with autoimmune diseases, potentially improving pregnancy outcomes in those with APS. As reported by Hooper et al., adding hydroxychloroquine to standard treatment regimens may enhance live birth rates in patients with obstetric APS [35].

Importantly, at the molecular level, anti-β2-glycoprotein I antibodies have been shown to induce tissue factor expression in endothelial cells and platelets via IRAK1 phosphorylation and NF-κB activation, thereby amplifying prothrombotic activity in the reproductive vasculature. This mechanism directly links immune dysregulation to thrombosis, with the procoagulant effect being attenuated by selective heparanase inhibition (RDS3337), suggesting a novel therapeutic target for APS-related pregnancy morbidity [55]. Autoimmune infertility has been increasingly linked to antiphospholipid antibodies, which interfere with reproductive processes by activating signaling pathways in endothelial and reproductive cells. Through lipid raft microdomains, these antibodies engage receptors such as TLR4, LRP6, and LRP8, disrupting normal vascular and cellular functions and fostering conditions that impair fertility [56,57].

Interestingly, the association between APL and infertility extends beyond recurrent pregnancy loss to include other obstetric complications such as preeclampsia and fetal growth restriction. A study by Manuck et al. indicated that women with APL are at increased risk for preeclampsia, as the antibodies can contribute to placental vascular complications, thereby affecting fetal growth and development [36]. Furthermore, Wang et al. detailed the implications of APL on preterm birth and the necessity of tailored prenatal care in affected individuals [58].

It is important to note that not all patients with APL will experience infertility or adverse pregnancy outcomes. The clinical manifestations can vary widely, and factors such as the presence of additional autoimmune conditions or genetic predispositions may also play essential roles. Studies indicate that genetic variations among individuals may affect the likelihood and severity of APS impact on reproductive health, warranting individualized assessment and management approaches in clinical practice.

Antiphospholipid antibodies are increasingly recognized as a crucial factor in infertility and recurrent pregnancy loss due to their propensity to induce thrombotic events and promote a pro-inflammatory state detrimental to pregnancy success. The interplay between autoimmune responses and reproductive health necessitates careful evaluation and management strategies for women facing infertility linked to these antibodies. Advances in diagnostics and therapeutic strategies hold promise for improving reproductive outcomes in this population, ultimately enhancing the quality of care provided to affected individuals.

## 8. Antithyroid Antibodies

Antithyroid antibodies, specifically those targeting thyroid peroxidase (anti-TPO) and thyroglobulin (anti-Tg), have garnered attention concerning their potential associations with infertility. Thyroid autoimmunity, particularly in conditions such as Hashimoto’s thyroiditis and Graves’ disease, has been linked to several reproductive outcomes, including delayed conception and recurrent pregnancy loss. This synthesis aims to elucidate the mechanisms by which antithyroid antibodies may influence fertility and outline clinical implications for affected individuals.

Research indicates that antithyroid antibodies may negatively impact ovarian function and overall reproductive health. Monteleone et al. proposed that elevated levels of anti-TPO antibodies can disrupt the ovarian follicle environment, potentially leading to impaired follicular development and reduced oocyte quality [6]. This hypothesis suggests that autoimmunity might interfere with the reproductive system, potentially resulting in infertility or reduced fertility in affected women.

The impact of thyroid autoantibodies extends beyond initial infertility challenges to possible complications during pregnancy. Rahnama et al. highlighted that anti-TPO antibodies may target reproductive tissues, including the endometrium and placenta, influencing implantation and early pregnancy viability [37]. These antibodies can present immunological challenges, potentially leading to pregnancy complications such as gestational hypertension, as suggested in women with existing thyroid disorders.

Assessing the link between infertility and thyroid autoimmunity has identified a need for early diagnosis and intervention. Birjandi et al. demonstrated a correlation between the presence of thyroid autoantibodies and altered thyroid hormone levels in women facing infertility, although they noted no direct link between infertility and other thyroid function tests [7]. Their findings underscore the necessity of screening for thyroid dysfunction in women presenting with infertility or recurrent pregnancy loss.

The implications of antithyroid antibodies also extend into ART. Studies indicate that these autoantibodies can adversely affect embryo quality and success rates in procedures such as IVF. For instance, Wu et al. found no significant differences in ovarian reserve indicators among women with thyroid autoantibodies, suggesting that the presence of these autoantibodies may not directly affect ovarian reserve but highlights the need for careful management of thyroid function in ART cycles [38].

Management strategies for women with antithyroid antibodies typically focus on normalizing thyroid function, often through interventions such as levothyroxine therapy. Treating underlying hypothyroidism can significantly enhance reproductive outcomes in women affected by thyroid autoimmunity, promoting better pregnancy results in subsequent attempts [39].

Moreover, the relationship between vitamin D and thyroid autoimmunity has emerged as a relevant consideration; deficiencies in vitamin D may be clinically associated with both thyroid dysfunction and reproductive issues [40]. Addressing such deficiencies through supplementation could have potential benefits for women with infertility linked to thyroid autoimmunity.

Considering the multifaceted relationship between antithyroid antibodies and infertility, it is crucial for clinicians to thoroughly evaluate thyroid function and autoimmune status in women presenting with infertility. Systems of care that integrate endocrinology, immunology, and reproductive health can improve the overall approach to supporting women facing these complexities. Clinical guidelines suggest that proactive management of antithyroid antibodies may play a vital role in optimizing fertility outcomes, particularly in at-risk populations.

Antithyroid antibodies represent a significant factor in the discourse on reproductive immunology. Their presence in women of reproductive age can profoundly influence fertility and pregnancy outcomes, emphasizing the need for integrated management approaches. As research continues to evolve, understanding the implications of thyroid autoimmunity in reproductive health remains central to addressing infertility.

## 9. Potential Role in Diagnostic

In the context of infertility diagnostics, the role of various antibodies has emerged as a vital area of research. These antibodies can be indicators of underlying immune factors that may inhibit conception or complicate pregnancy. Specifically, the presence of autoimmune and infection-related antibodies has been shown to contribute significantly to infertility diagnoses. This review discusses the potential diagnostic applications of antithyroid, antisperm, and APL, along with antibodies linked to infections such as Chlamydia trachomatis.

Antithyroid antibodies, particularly anti-TPO and anti-Tg antibodies, have been linked to female infertility. They may disrupt normal thyroid function, which is critical for regular menstrual cycles and ovulation. Autoimmune thyroid disease, characterized by these antibodies, can lead to conditions like subclinical hypothyroidism, which adversely affects reproductive health [41]. Investigations indicate that measuring levels of antithyroid antibodies in infertile women may be crucial for proper diagnosis and to guide treatment strategies aimed at optimizing thyroid health during conception efforts [59].

Autoimmune factors, including ASA, have also been central to understanding infertility. ASA can inhibit sperm motility and impair fertilization processes, significantly lowering the chances of conception [24]. Their presence can be detected through serological tests, such as the mixed antiglobulin reaction test, which is a method to identify the antibodies affecting sperm function [23]. Testing for ASA has gained recommendation as a formal part of the infertility workup, especially before ART like IVF [60]. The presence of these antibodies can also indicate a need for careful manipulation of gametes in ART to prevent adverse effects on developmental outcomes.

Antiphospholipid antibodies have demonstrated a significant correlation with reproductive failure, especially recurrent pregnancy loss and infertility. The measurement of APL, such as anticardiolipin and anti-β2 glycoprotein I antibodies, can help identify individuals at risk for complications during pregnancy. These antibodies can cause thrombosis in placental arteries and adversely affect endometrial receptivity and trophoblast invasion, thus contributing to infertility or adverse reproductive outcomes [61]. Recent data favor the inclusion of APL testing within infertility assessments, particularly when unexplained infertility is diagnosed or when there is a history of pregnancy loss [62].

The implications of infection-related antibodies in infertility diagnosis are particularly evident in infections like Chlamydia trachomatis. This organism is recognized as a common cause of pelvic inflammatory disease, which can lead to tubal factor infertility due to scarring or blockage of the fallopian tubes. Research has indicated that measuring antibodies against chlamydia can serve as an important biomarker in diagnosing infertility and identifying women who might benefit from further evaluation through hysterosalpingography (HSG) and laparoscopy [63,64]. This approach highlights the diagnostic value of serology in guiding appropriate clinical interventions.

In addition to these specific antibodies, emerging research emphasizes the importance of a broader immunological assessment in the diagnostic process for infertility. For instance, evaluations of cytokine profiles and T cell subsets can provide insights into the immunological landscape affecting reproductive health. Cytokine profiling of serum, follicular fluid, or endometrial secretions can reveal pro-inflammatory or anti-inflammatory immune imbalances that may influence implantation success. [65]. Assessment of endometrial NK cell density and activation status, including proportions of CD56^+^ and CD16^+^ cells, has been linked to implantation failure and recurrent pregnancy loss. Furthermore, circulating or endometrial-derived exosomal biomarkers, which carry immunomodulatory molecules, are showing potential as early indicators of implantation failure risk.

These novel tools, alongside conventional serological markers, have shown promise in stratifying patients with suspected immunological infertility and in guiding personalized therapeutic strategies. Incorporating both traditional and emerging immunodiagnostic approaches may enhance the precision of infertility assessment and improve clinical outcomes.

A variety of diagnostic techniques, including serological tests and imaging modalities, can be leveraged to assess immune and infectious factors contributing to infertility. This multifaceted approach facilitates early identification of any contributing pathological conditions, potentially improving treatment outcomes. By incorporating these biomarkers into routine evaluations, clinicians can enhance early diagnosis, mitigate risks, and tailor fertility treatments to the individual needs of patients.

Antibody testing plays a pivotal role in infertility diagnostics, offering insights into both immunological parameters and infection-related complications. The strategic integration of tests for antibodies—including antithyroid, antisperm, and antiphospholipid antibodies—along with infectious agents such as Chlamydia trachomatis and newer immunoprofiling tools can significantly enhance our understanding of infertility and guide individualized therapeutic strategies. Diagnostic tools for different types of antibodies are shown in the Table 2.

## 10. Treatment Modalities and Management of Autoimmune Infertility

### 10.1. Immunomodulatory Therapies

For women with autoimmune antibodies contributing to infertility, especially those with conditions like APS or autoimmune thyroid disease, immunomodulatory therapies are often integral to management. Tanaçan and Beksaç reported that individualized approaches such as low-dose methylprednisolone and low-dose aspirin can be beneficial for managing autoimmune disorders in infertile patients [66]. These treatments aim to modulate the immune response, potentially improving the likelihood of successful pregnancies.

Intravenous immunoglobulin therapy represents another promising treatment for women experiencing immune-related infertility. Studies highlight its role in modulating the immune response and enhancing pregnancy outcomes, particularly in cases with recurrent pregnancy loss due to autoimmune factors [29]. A report detailing successful pregnancies for women undergoing IVF noted that immunomodulation therapy, including plasmapheresis and glucocorticoids, had favorable outcomes in women believed to have antibodies against human chorionic gonadotropin (anti-hCG) autoimmunity, underscoring the need for such therapies in autoimmune-related infertility [29].

### 10.2. Hormonal Replacement Therapy

For patients with thyroid-related autoimmune conditions, thyroid hormone replacement therapy presents a critical aspect of managing infertility. Grigoriadis et al. suggest that proper management of thyroid autoantibodies and relevant hormonal imbalances can improve reproductive outcomes in women undergoing ART [67]. Regular monitoring and tailored adjustments of thyroid hormones, such as levothyroxine, are important for enhancing ovulation and fertilization rates, particularly in patients with subclinical hypothyroidism.

Similarly, the management of hormonal deficiencies in conditions like primary ovarian insufficiency can significantly impact treatment plans. Hormone replacement therapy remains an effective approach to alleviate symptoms and promote a healthier reproductive environment for women with autoimmune-induced infertility. Ensuring optimal hormonal levels may improve their chances of conception.

### 10.3. Addressing Infections and Inflammatory Conditions

Furthermore, addressing infections such as Chlamydia trachomatis is crucial, as it significantly contributes to tubal factor infertility due to scarring and adhesions resulting from pelvic inflammatory diseases [68]. Testing for antibodies against infections can complement a comprehensive infertility workup and guide appropriate treatments, including antibiotics, to mitigate factors interfering with fertility.

Management of chronic inflammatory conditions is another key area. In cases where autoimmune disorders contribute to infertility, anti-inflammatory medications may reduce systemic inflammation and improve reproductive function. For instance, corticosteroids have been used to suppress excessive immune reactions, thereby reducing the risk of complications during conception efforts [69].

### 10.4. Lifestyle Interventions

Lifestyle factors cannot be overlooked, as they play a crucial role in reproductive health. Women diagnosed with autoimmune diseases may benefit from dietary adjustments, stress management, and other lifestyle changes [70]. A well-balanced and nutrient-rich diet supports immune and overall health, which may subsequently enhance fertility. Research is exploring the impact of micronutrient deficiencies on autoimmune conditions and their potential relationship with infertility. Adequate levels of vitamins such as D, B12, and folate could be vital in improving reproductive outcomes for women with autoimmune conditions [6].

### 10.5. Care Coordination

Coordination among multidisciplinary medical professionals—including reproductive endocrinologists, immunologists, and lifestyle coaches—ensures comprehensive care tailored to women’s specific autoimmune and fertility needs. Effective communication within a healthcare team promotes holistic interventions that are rooted in a thorough understanding of the patient’s medical history, reproductive goals, and autoimmune status [71].

### 10.6. Emerging Therapies

Research continues to explore novel therapeutic options for the management of autoimmune infertility. The potential role of stem cell therapies is being investigated in relation to treating primary ovarian insufficiency and other autoimmune-related fertility issues [72]. Studies on the efficacy of autologous stem cell transplantation to restore ovarian function indicate promising results; however, further investigation is needed to clarify their long-term implications and effectiveness [73].

In summary, the management of autoimmune infertility necessitates a comprehensive understanding of the underlying immunological factors contributing to fertility challenges. Treatment modalities encompass a combination of immunomodulatory therapies, hormonal management, addressing inflammatory and infectious concerns, lifestyle improvements, and coordinated care efforts. By establishing personalized management plans that consider both autoimmune and infertility concerns, clinicians can enhance the likelihood of successful reproductive outcomes for affected women. Therapeutic strategies targeting autoimmune infertility are shown in Table 3.

## 11. Conclusions

Autoimmune infertility represents a significant but often overlooked component of reproductive dysfunction. The current body of evidence reveals that a wide range of autoantibodies—including ANA, ASA, AOA, AEA, APL, and antithyroid antibodies—can detrimentally affect fertility in both women and men. These antibodies may interfere with critical reproductive processes such as gamete interaction, implantation, hormonal balance, and placental development. Importantly, many of these immunological alterations occur in the absence of overt autoimmune disease, which necessitates heightened clinical vigilance.

The presence of ANA has been repeatedly linked to diminished oocyte quality, impaired embryo implantation, and recurrent pregnancy loss in women undergoing ART [3,10]. Similarly, APL contribute to recurrent miscarriage and implantation failure, largely through their pro-thrombotic and pro-inflammatory actions [5]. ASA in males have been associated with decreased sperm motility and impaired fertilization, particularly in natural conception scenarios [8]. Furthermore, autoimmune thyroid disease, even when biochemically euthyroid, poses a risk for subfertility and miscarriage, reinforcing the need for early detection and hormone optimization [6].

One of the key takeaways of this review is the necessity of incorporating immunological assessment into the diagnostic work-up of infertility, particularly in cases labeled as “unexplained.” Testing for ANA, APL, ASA, and thyroid antibodies—alongside a thorough autoimmune history—can unveil previously unidentified etiologies and guide more targeted interventions.

Therapeutically, evidence supports the use of immunomodulatory strategies in selected patients. Low-dose corticosteroids, aspirin, and heparin have been employed in women with autoimmune infertility, particularly those with APS or positive ANA [15,46]. Additionally, hydroxychloroquine has emerged as a promising agent in improving ART outcomes among women with autoantibodies [4]. Hormonal correction in cases of thyroid dysfunction or premature ovarian insufficiency remains essential.

Lifestyle interventions, such as optimizing vitamin D and folate levels, managing stress, and treating chronic inflammation, may also enhance immune regulation and fertility potential. Multidisciplinary care involving reproductive endocrinologists, immunologists, and general practitioners is indispensable for individualized and holistic patient management.

Looking ahead, significant research gaps remain in understanding autoimmune infertility. While the pathogenic roles of various autoantibodies are increasingly recognized, their exact mechanisms, predictive value, and optimal therapeutic strategies require clarification. Future work should focus on standardizing diagnostic criteria, validating biomarker panels, and integrating immunoprofiling to better identify patients who may benefit from targeted interventions. Large, prospective trials are needed to assess the effectiveness and safety of immunomodulatory therapies, as well as their long-term reproductive outcomes.

Emerging treatments also offer promising directions. Targeted receptor antagonists and selective heparanase inhibitors—shown in preclinical studies to have immunomodulatory and antithrombotic effects—could provide innovative options, particularly in APS-related infertility. Further translational research on these and other novel agents may help bridge laboratory findings with clinical practice, leading to more effective, mechanism-based therapies.

In conclusion, reproductive immunology continues to expand as a vital field in infertility research and clinical practice. Autoimmune mechanisms may underlie a substantial proportion of otherwise unexplained infertility and should not be neglected. As diagnostic tools improve and targeted therapies become more accessible, clinicians are better equipped to address immune-related infertility and improve reproductive outcomes for affected individuals. Future research should focus on refining diagnostic criteria, exploring novel immunotherapies, and validating treatment protocols to ensure that patients with autoimmune infertility receive evidence-based, personalized care. Summary of antibodies involved in autoimmune infertility is presented in Table 4.

## Figures and Tables

**Table 1:** Infertility in relation to autoimmunity.

Autoantibodies	Reference	Main Results
Antinuclear antibodies (ANA)	Chen et al. (2023) [3], Guo et al. (2023) [4], Li et al. (2020) [10], Wu et al. (2022) [14], Mitic et al. (2019) [15], Cavalcante et al. (2020) [13], Fan et al. (2017) [16], Moyer & Edens (2024) [17]	ANA are associated with diminished oocyte quality, increased miscarriage rates, and IVF failure. They may interfere with implantation and embryo development. Treatments like corticosteroids and hydroxychloroquine have shown improvements in reproductive outcomes.
Antisperm antibodies (ASA)	Silva et al. (2021) [8], Sciorio et al. (2025) [18], Bozhedomov et al. (2014) [19], Wang et al. (2012) [20], Kamphorst et al. (2021) [21], Gupta et al. (2022) [22], Shibahara et al. (2021, 2022) [23,24]	ASA impair sperm motility, capacitation, and the acrosome reaction, reducing chances of natural conception. Often observed in varicocele, post-vasectomy, and infection. ICSI helps bypass ASA effects. Diagnostic testing like MAR is essential in infertility workups.
Antiendometrial antibodies (AEA)	Bobak et al. (2014) [25], Menon et al. (2016) [26], Caccavo et al. (2011) [27]	AEA disrupt endometrial receptivity, impair implantation, and are linked to recurrent pregnancy loss. Frequently associated with endometriosis and chronic infections. Anti-laminin-1 antibodies indicate altered implantation environments.
Antiovarian antibodies (AOA)	Luborsky et al. (2011) [28], Müller et al. (2016) [29], Abdullah (2020) [30], Khadhim et al. (2015) [31], Chen (2022) [32], Abood & Hathal (2021) [33]	AOA are implicated in premature ovarian insufficiency and disrupted folliculogenesis. They may co-occur with anti-FSH antibodies and PCOS, leading to ovulatory dysfunction. Immunosuppressive treatment is under investigation for fertility improvement.
Antiphospholipid antibodies (APL)	Poindron et al. (2011) [11], Kirovakov et al. (2024) [34], Schreiber et al. (2017) [5], Hooper et al. (2023) [35], Manuck et al. (2010) [36]	APL are strongly linked to recurrent pregnancy loss, preeclampsia, and implantation failure due to placental thrombosis and inflammation. Treatments with aspirin, heparin, and hydroxychloroquine improve outcomes in APS patients undergoing ART.
Antithyroid antibodies (anti-TPO, anti-Tg)	Monteleone et al. (2011) [6], Birjandi et al. (2021) [7], Rahnama et al. (2021) [37], Wu et al. (2021) [38], Oiwa et al. (2019) [39], Pankiv (2020) [40], Zohora et al. (2022) [41]	Antithyroid antibodies are associated with impaired oocyte quality and endometrial receptivity. Even euthyroid women may experience reduced fertility. Hormone replacement (e.g., levothyroxine) and vitamin D optimization improve reproductive outcomes.

**Table 2:** Conventional and emerging immunodiagnostic tools for infertility.

Diagnostic Tool	Sample Type	Clinical Relevance
Antinuclear antibodies (ANA)	Serum	Detects systemic autoimmunity that may impair reproductive outcomes
Antiphospholipid antibodies (APL: anticardiolipin, anti-β2GPI)	Serum	Associated with thrombosis, impaired implantation, recurrent pregnancy loss
Antisperm antibodies (ASA)	Serum, semen	Impair sperm motility and fertilization, important before ART
Antithyroid antibodies (anti-TPO, anti-Tg)	Serum	Linked to thyroid dysfunction affecting ovulation and pregnancy maintenance
Chlamydia trachomatis antibodies	Serum	Marker of prior infection, risk factor for tubal factor infertility
Cytokine profiling	Serum, follicular fluid, endometrial secretions	Identifies inflammatory/anti-inflammatory balance influencing implantation
Endometrial NK cell density & activation (CD56^+^, CD16^+^)	Endometrial biopsy	Predicts implantation success; abnormal levels linked to recurrent pregnancy loss
Exosomal biomarkers	Serum, endometrial fluid	Carry immune-regulatory molecules; potential early predictor of implantation failure
T cell subset analysis (Th1/Th2 balance, regulatory T cells)	Peripheral blood, endometrial tissue	Reflects immune tolerance status relevant to embryo implantation

**Table 3:** Therapeutic strategies targeting autoimmune infertility.

Antibody	Therapeutic Class	Treatment/Intervention	Evidence Level *	Reference(s)
ANA (Antinuclear antibodies)	Corticosteroids, IVIG, Antimalarials	Low-dose methylprednisolone, IVIG, prednisone + aspirin, hydroxychloroquine	III–IV	[10,12,13]
APL (Antiphospholipid antibodies)	Antiplatelet, Anticoagulant, Corticosteroids, Antimalarials	Low-dose aspirin, heparin, corticosteroids, hydroxychloroquine	II–III	[35,36,61,62]
AOA (Anti-Ovarian antibodies)	Corticosteroids, Hormonal therapy	Glucocorticoids, hormone replacement therapy (HRT)	IV	[33,68]
Anti-hCG antibodies	Corticosteroids, IVIG, Plasmapheresis	Glucocorticoids, IVIG, plasmapheresis	IV	[29]
Anti-TPO antibodies	Hormonal therapy	Levothyroxine therapy, thyroid hormone optimization	III	[37,38,41,68]
Anti-sperm antibodies	Corticosteroids, Immunomodulation, ART	Glucocorticoids, IVIG, ASA, hydroxychloroquine, assisted reproductive techniques	III	[8,9,22,59]
Anti-LH/Anti-FSH antibodies	Hormonal therapy	HRT, ovulation induction	IV	[33]
Anti-Chlamydia antibodies	Antibiotics	Targeted antimicrobial therapy	III	[63,64]
Anti-laminin-1 antibodies	Immunomodulation	IVIG, glucocorticoids	IV	[27]
Anti-mesothelin antibodies	Immunomodulation	IVIG, immunotherapy	IV	[28]

* Evidence levels: Level I—systematic review/meta-analysis of RCTs; Level II—single RCT or well-designed controlled trial; Level III—well-designed cohort or case-control study; Level IV—case series or poor-quality cohort/case-control; Level V—expert opinion.

**Table 4:** Summary of antibodies involved in autoimmune infertility.

Antibody	Primary Mechanism of Action	Potential Effects	Clinical Consequences	Potential Therapies
Antiphospholipid antibodies (APL)	Bind to phospholipid-binding proteins, promote thrombosis, activate complement, induce local inflammation in uterus/placenta	Placental vascular thrombosis, impaired implantation, inflammation	Recurrent pregnancy loss, preeclampsia, fetal growth restriction, preterm birth	Low-dose aspirin, heparin, corticosteroids (in select cases)
Antinuclear antibodies (ANA)	Bind to nuclear antigens, trigger systemic inflammation	Impaired oocyte quality, disrupted embryo development, implantation failure	Unexplained infertility, recurrent pregnancy loss	Immunomodulatory therapy (corticosteroids, hydroxychloroquine), tailored fertility protocols
Antisperm antibodies (ASA)	Bind to sperm antigens, block motility, impair capacitation and acrosome reaction	Reduced sperm transport, impaired fertilization	Male or immunologic infertility	Corticosteroids, assisted reproductive technologies (ART)
Anti-oocyte antibodies (AOA)	Target oocyte antigens, impair oocyte maturation and function	Reduced fertilization rates	Poor response to ART, diminished ovarian reserve	Immunosuppression, ART with oocyte donation in severe cases
Antiendometrial antibodies (AEA)	Target endometrial tissue antigens (e.g., laminin-1)	Altered endometrial receptivity, local inflammation	Implantation failure, recurrent pregnancy loss	Corticosteroids, immunomodulatory agents
Antithyroid antibodies	Bind to thyroid antigens, alter thyroid hormone metabolism	Thyroid dysfunction, systemic inflammation	Menstrual irregularities, miscarriage, infertility	Thyroid hormone replacement, immunomodulation
Antichlamydia antibodies	Immune response to Chlamydia trachomatis antigens	Tubal scarring, inflammation	Tubal factor infertility	Antibiotics for active infection, ART if tubal damage is irreversible
Anti-fertilization antigen (FA-1) antibodies	Block sperm–oocyte binding	Fertilization failure	Infertility despite normal gametes	ART (ICSI), immunosuppression in select cases

## Data Availability

No new data were created or analyzed in this study. Data sharing is not applicable to this article.
